# Genetics and pathophysiology of mitral valve prolapse

**DOI:** 10.3389/fcvm.2023.1077788

**Published:** 2023-02-16

**Authors:** Constance Delwarde, Romain Capoulade, Jean Mérot, Solena Le Scouarnec, Nabila Bouatia-Naji, Mengyao Yu, Olivier Huttin, Christine Selton-Suty, Jean-Marc Sellal, Nicolas Piriou, Jean-Jacques Schott, Christian Dina, Thierry Le Tourneau

**Affiliations:** ^1^Nantes Université, CHU Nantes, CNRS, INSERM, L’institut du Thorax, Nantes, France; ^2^INSERM, PARCC, Université Paris Cité, Paris, France; ^3^Human Phenome Institute, Fudan University, Shanghai, China; ^4^Service de Cardiologie, Institut Lorrain du Coeur et des Vaisseaux Louis Mathieu, CHU de Nancy, Nancy, France

**Keywords:** mitral valve prolapse, myxomatous disease, genetic, pathophysiology, animal models, Filamin A, dachsous

## Abstract

Mitral valve prolapse (MVP) is a common condition affecting 2–3% of the general population, and the most complex form of valve pathology, with a complication rate up to 10–15% per year in advanced stages. Complications include mitral regurgitation which can lead to heart failure and atrial fibrillation, but also life-threatening ventricular arrhythmia and cardiovascular death. Sudden death has been recently brought to the forefront of MVP disease, increasing the complexity of management and suggesting that MVP condition is not properly understood. MVP can occur as part of syndromic conditions such as Marfan syndrome, but the most common form is non-syndromic, isolated or familial. Although a specific X-linked form of MVP was initially identified, autosomal dominant inheritance appears to be the primary mode of transmission. MVP can be stratified into myxomatous degeneration (Barlow), fibroelastic deficiency, and Filamin A-related MVP. While FED is still considered a degenerative disease associated with aging, myxomatous MVP and FlnA-MVP are recognized as familial pathologies. Deciphering genetic defects associated to MVP is still a work in progress; although *FLNA*, *DCHS1*, and *DZIP1* have been identified as causative genes in myxomatous forms of MVP thanks to familial approaches, they explain only a small proportion of MVP. In addition, genome-wide association studies have revealed the important role of common variants in the development of MVP, in agreement with the high prevalence of this condition in the population. Furthermore, a potential genetic link between MVP and ventricular arrhythmia or a specific type of cardiomyopathy is considered. Animal models that allow to advance in the genetic and pathophysiological knowledge of MVP, and in particular those that can be easily manipulated to express a genetic defect identified in humans are detailed. Corroborated by genetic data and animal models, the main pathophysiological pathways of MVP are briefly addressed. Finally, genetic counseling is considered in the context of MVP.

## Introduction

Mitral valve prolapse (MVP) is the most common form of valve disease, occurring in 2–3% of the general population, and up to 5–6% when prodromal or minimal forms are included (1, 2). MVP is classically defined as a 2 mm or greater displacement in systole of the tip or the body of valve leaflet above the plane of the mitral annulus in parasternal long-axis view on echocardiography (1). Although the course of MVP is most often benign, it can lead to the development of severe mitral regurgitation (MR), complicated by heart failure and supraventricular arrhythmia, but MVP can also be associated to ventricular rhythm disorders, and sometimes sudden death. MVP is frequent in some syndromic diseases such as Marfan’s syndrome, but is generally encountered in isolated, non-syndromic forms (3). Clinicians distinguish classically two main types of MVP, fibroelastic degeneration (FED) which occurs in the elderly, and myxomatous degeneration also called Barlow’s disease which develops earlier in the lifespan (4). Differences between these two main types are based on macroscopic examination during surgery, histology, but also on echocardiographic findings. Myxomatous and FED MVP were originally described by the team of Carpentier et al. following surgical observation, reinforced by histological differences. Briefly, FED MVP is characterized by thin, translucent leaflets with dystrophy (elongation and thickening) of the prolapsing segment, usually related to rupture of a chordae tendinae. In contrast, in myxomatous MVP, leaflet dystrophy is diffuse with thickening, elongation, and prolapse of all segments of the anterior and posterior leaflets, dilatation of the mitral annulus, and frequent disjunction (1, 4, 5). In addition, minor or prodromal forms of MVP characterized by limited mitral leaflet dystrophy but with a leaflet displacement of less than 2 mm have recently been recognized, and should be considered in familial or genetic approaches to MVP (2, 6–8).

The identification of rare genetic variants with a strong effect or common variants with a weak effect promoting the development of MVP allow us to approach the underlying pathophysiological mechanisms associated with this condition. Further, generation of transgenic animal models help to better characterize the phenotypic expression of MVP in its different forms and as an ultimate goal, to identify therapeutic targets that will allow to slow down or stop the evolution of the disease.

## Familial clustering and mode of inheritance

As early as 1966, Barlow and Bosman (9) reported familial forms of late-systolic murmur and click suggesting that a hereditary factor could sometimes be involved in the development of mitral valve (MV) dysfunction. In 1968, Monteleone et al. reported a familial clustering of MVP (10) in a small family with disease distribution consistent with an X-linked mode of inheritance. In a systematic familial screening, Devereux et al. (11) confirmed in the early 1980s the familial clustering of MVP, and from 45 probands a distribution of disease consistent with an autosomal dominant condition. In this series, MVP was diagnosed by echocardiography in 30% of first-degree relatives with a number of affected individuals within the range predicted for autosomal dominant inheritance. Further, at least one first-degree relative was affected in 60% of families screened. In addition, MVP was more frequent in women compared with men (41 vs. 19%), and in adults compared to children. Hence MVP was seen the commonest mendelian cardiovascular abnormality in humans. At the end of the 1990s, several large families of MVPs were identified and analyzed, and several loci were identified on the X chromosome and autosomal chromosomes, thus demonstrating genetic heterogeneity. In addition to familial clustering and genetic inheritance confirmation, familial screening evidenced the common finding of prodromal forms of MVP (minimal billowing, posterior leaflet lengthening and anterior displacement of coaptation, restrictive motion of leaflets), suggesting that MVP encompasses a more complex phenotypic expression than previously thought (2, 7). It is noteworthy that prodromal forms can be the only expression of a MVP familial disease (2, 7), and prodromal forms in parents can increase the risk of MVP development in offsprings (6, 8). Further, Delling et al. have shown in the Framingham Heart Study and Swedish population that clustering of MR exists in the community (12), supporting a genetic susceptibility to primary and non-primary MR (12, 13).

To conclude, apart from sporadic forms, myxomatous MVP is usually a primary, dominantly inherited condition with incomplete and age-dependent penetrance, but can also be an X chromosome inherited disease.

## Genetics of MVP

Since MVP is a common disease, it is likely that common genetic variants account for part of the genetic component of MVP. However, as with most common diseases, a significant subset of MVP cases clusters within families following a Mendelian inheritance pattern, predicting that rare variants having strong effects can also lead to disease development. In this context, classical linkage analysis was first used to map candidate regions of the genome for MVP, allowing disease gene mapping at Xq28, 16p11, 11p15, and 13q31-q32 (7, 14–16), and the eventual identification of rare genetic variants as a cause of familial MVP.

More recently, whole exome and whole genome sequencing, offering deep sequencing, are becoming the standard for gene discovery and clinical molecular diagnosis. In addition, high-throughput genomic technologies have enabled the implementation of genome-wide association studies (GWAS) in search of common genetic variants predisposing to common diseases. These recent technologies have eased the deciphering of the genetic background of MVP. The main genetic defects associated with syndromic and non-syndromic forms of MVP, according to current knowledge, are summarized in Table 1.

**TABLE 1:** Genetic anomalies associated with mitral valve prolapse in humans.

	Gene or chromosome defect	Defect localization/Mechanism
**Syndromic MVP**		
Trisomy 18, 13, 15	Chr 18, 13, 15	–
Down syndrome	Chr 21	–
Marfan syndrome	FBN1 Chr 15 TGFBR1 Chr 9 TGFBR2 Chr 3	TGFβ pathway
MASS	FBN1 Chr 15?	TGFβ pathway?
Loeys-Dietz syndrome	TGFBR1 Chr 9 TGFBR2 Chr 3 SMAD3 Chr 15 SMAD2 Chr 18 TGFβ2 Chr 1 TGFβ3 Chr 14	TGFβ pathway
Juvenile polyposis syndrome	SMAD4 Chr18 BMPR1A Chr10	TGFβ pathway
Aneurysms-osteoarthritis syndrome	SMAD3 Chr 15	TGFβ pathway
Ehlers-Danlos syndrome	Multiples genes, some forms with valve dystrophy	ECM
Osteogenesis imperfecta	COL1A1 Chr 17	ECM
Williams-Beuren syndrome	ELN Chr 7	ECM
Pseudoxanthoma elasticum	MRP6 (ABCC6) Chr 16	ECM
BDCS or FTH syndromes	SH3PXD2B Chr 5	Podosomes/cell migration
Larsen-like syndrome	B3GAT3 Chr 11	ECM/glycosaminoglycans
Sinus node dysfunction, arrhythmias, LVNC	HCN4 Chr 15	Ionic channel/heart development
**Non-syndromic MVP**		
FilaminA-MVD/MVP	FLNA Chr X	Mechanotransduction, ciliation
Dachsous1-MVP	DCHS1 Chr 11	Cell migration and polarity, ciliation
DZIP1-MVP	DZIP1 Chr 13	Ciliation

BDCS, Borrone dermato-cardio-skeletal; ECM, extracellular matrix; FTH, Frank-Ter Haar; LVNC, left ventricular non-compaction; MASS, mitral aorta skeleton and skin phenotype.

### Syndromic forms of MVP

Mitral valve prolapse has been observed in many syndromic disorders, this association being sometimes related to chance because of the prevalence of MVP in the general population (17), or to inadequate or unclear diagnostic criteria of MVP. Indeed, some disorders previously believed to have an increased prevalence of MVP were based on outdated diagnostic criteria, or, evidences of a true pathological association were absent. In addition, many congenital heart diseases can present some degree of MV dystrophy that can impose for a MVP. Hence, we will therefore focus the present review on the most common syndromic disorders for which there are solid arguments in terms of prevalence and/or pathophysiological data.

#### Marfan syndrome

**FIGURE 1:**
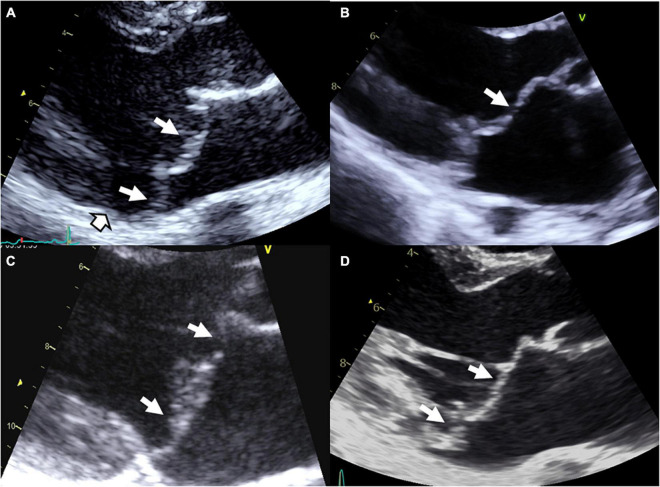
Syndromic and non-syndromic mitral valve prolapse/dystrophy. **(A)** Marfan syndrome with bileaflet myxomatous MVP, **(B)** Williams–Beuren syndrome with a history of operated supra-valvular aortic stenosis, mild billowing/prolapse of the anterior leaflet and restrictive motion of the posterior leaflet with moderate MR, **(C)** Filamin A MVP/dystrophy, and **(D)** bileaflet myxomatous MVP diagnosed after a resuscitated sudden death inaugurating the disease. White arrows: Prolapse/billowing, black and white head arrow: Mitral annular disjunction common in arrhythmic MVP.

Marfan syndrome is an autosomal dominant connective condition that affects 1 in 5,000 individuals. Marfan syndrome is primarily due to variants in the gene *FBN1*, encoding the connective protein fibrillin-1, that alter the transforming growth factor β (TGFβ) pathway. Although most variants are inherited, 25% of patients develop the disease as a result of a *de novo* mutation. Mutations in *TGFBR1* or *TGFBR2* genes have been reported in some patients with atypical presentation but nevertheless consistent with Marfan syndrome. In case of MVP (Figure 1A), Marfan syndrome diagnosis, based on revised Ghent criteria (18), is crucial because of the risk of aortic dissection. The diagnosis of Marfan syndrome should be evoked in the presence of aortic root dilation, personal or familial aortic dissection/aneurysm rupture, or in the presence of systemic signs (18). In Marfan syndrome, MVP is common and can be the first clinical or echocardiographic finding (19). Penetrance and prevalence of MVP increase over time from 43% at 30 years to 77% at the age of 60 (20). Referral to surgery for isolated severe MR is uncommon in Marfan syndrome, but mitral surgery is performed in up to 13% of patients with Marfan syndrome at the age of 60. However, outcome is dominated by the aorta risk of rupture or dissection. Next to the typical Marfan syndrome, the MASS syndrome is a marfanoid syndrome that affects mitral, aorta, skeleton, and skin (MASS) but doesn’t fulfill the Ghent criteria. Genetic background of MASS is unknown but *FBN1* mutations have been also identified in some patients. The prevalence of MVP seems high in this mild form of Marfan syndrome (17). Transforming growth factor β signaling alteration has been associated with the development of MVP in Marfan syndrome but also in Loeys-Dietz syndrome.

#### Loeys-Dietz syndrome

Loeys-Dietz syndrome (LDS) is a rare connective tissue disorder, with autosomal dominant inheritance, that arises from variants altering the TGFβ signaling pathway. The 5 different types are LDS types 1 and 2 as a result of mutations in *TGFBR1* and *TGFBR2*, respectively, LDS type 3 which results of mutations in *SMAD3*, whereas LDS type 4 and 5 results of mutations in *TGFB2* and *TGFB3*, respectively (21). Finally, LDS can also result from mutations in *SMAD2*. Mitral valve prolapse seems more frequent in LDS types 4 and 5 (17–21%) than in types 1, 2, and 3 (5–13%). Compare to Marfan syndrome carrying *FBN1* mutation, MV involvement is less frequent in LDS type 2: MVP was found in 21 vs. 45% of patients (*P* = 0.001), MR in 35 vs. 56% (*p* < 0.0001) and referral to MV surgery was rare in *TGFBR2* patients (19).

#### Other syndromes

Other syndromes associated with MVP involving the TGFβ pathway are the juvenile polyposis syndrome (*SMAD4*: Mothers against decapentaplegic homolog 4 or SMAD family member-4 and *BMPR1A*: Bone morphogenetic protein receptor, type 1A), and the aneurysm-osteoarthritis syndrome (Mutations in *SMAD3*: SMAD family member-3).

Borrone dermato-cardio-skeletal (BDCS) and Frank-Ter Haar (FTH) syndromes are autosomal-recessive disorders characterized by cutaneous, cardiovascular (with MVP), skeletal and other abnormalities owing to homozygote mutations in the *SH3PXD2B* gene (encoding the protein SH3 and PX domains 2B).

Finally, MVP have been reported with increased prevalence in aneuploidies including Down’s syndrome, Williams–Beuren syndrome (Figure 1B), Ehlers–Danlos syndrome, osteogenesis imperfecta, pseudoxanthoma elasticum, Stickler syndrome, Larsen-like syndrome, fragile X syndrome, and polycystic kidney disease, but data regarding these associations remain less convincing (3, 17).

### Non-syndromic MVP

Non-syndromic form of MVP is the most common condition of MVP. Non-syndromic MVP is a degenerative disease, worsening overtime (2) and associated with macroscopic, echocardiographic and histological modifications of MV apparatus (1, 4, 5). Non-syndromic MVP can be grossly stratified in myxomatous degeneration, fibro-elastic loss, and Filamin A-related MVP (Figure 1C) (3). Thanks to the recent identification of gene defects, MVP genotype-phenotype relationship refinement started, with the description of the unique Filamin A-MVP phenotype (2), and the identification of prodromal forms of MVP (2, 7, 8).

#### Filamin A (FLNA)

The first genetic defect of a non-syndromic MVP has been linked to *FLNA* variants in families with X-linked inheritance (2, 14, 22, 23). In the absence of extra-cardiac manifestations Filamin A-MVP is regarded a non-syndromic disease, but it is well known that some FLNA variants in other regions of the gene can cause syndromic diseases (2, 3, 23). The *FLNA* gene, located on chromosome X in the Xq28 region, encodes actin-binding protein that crosslinks actin filaments and links actin filaments to membrane glycoproteins. The penetrance of the disease is complete in men and incomplete in women. Filamin A-MVP phenotype (Figure 1C) associates both developmental and degenerative alterations of the MV apparatus and frequent polyvalvular disease in men. On histology, Filamin A-MVP demonstrates diffuse proteoglycan accumulation (2, 24) consistent with a myxomatous-like MVP but, in addition to prolapse in systole, leaflet motion is paradoxically restricted in diastole (2), consistent with papillary muscle and chordae development aberrations (2, 24, 25) and some degree of leaflet fibrosis and stiffening. Mitral annulus disjunction was not evidenced in these patients, and outcome is characterized by a higher rate of valve surgery (mitral and aortic) in men as compared to women, but also to classical bileaflet myxomatous MVP (2).

#### Dachsous 1 (DCHS1)

A loss-of-function mutation in *DCHS1* (dachsous cadherin-related 1, on chromosome 11 at the 11p15 location) gene, which segregates with myxomatous MVP in a large family pedigree, was recently diagnosed as a cause of MVP (26). Two other mutations were subsequently identified. *DCHS1* encodes a protein of the cadherin superfamily involved in calcium-dependent cell-cell adhesion. In mouse models with decreased expression of *DCHS1*, anterior and posterior mitral leaflets are enlarged and show myxomatous degeneration and prolapse, which could be traced back to developmental errors in valve morphogenesis. In addition, mutated mice and zebrafish exhibit abnormal planar cell polarity architecture of the valve matrix, and mitral leaflets show disorganized valvular interstitial cells (VICs), supporting these processes as etiological underpinnings for the disease.

#### DZIP1

Recently, a primary cilia gene, *DZIP1* (DAZ interacting zinc finger protein 1, on chromosome 13 at the 13q31-q32 location), has been identified as a causal gene for non-syndromic autosomal dominant MVP in humans. A deleterious missense mutation in *DZIP1* segregates with myxomatous MVP in a large family pedigree (27). Experimental findings reveal that myxomatous degeneration may occur through altered developmental pathways that involve both ciliogenesis and β-catenin signaling. Matrix metalloproteases were up-regulated in mitral leaflets with subsequent proteolysis of extra-cellular matrix (ECM) collagen and elastin, and myxomatous phenotype (28). In addition, a significant reduction in MV primary cilia length has been demonstrated in *Dchs1* and *FlnA* knockout (KO) mice (27). Hence, these findings support the hypothesis that MVP can be caused by abnormal primary cilia function.

### Common risk alleles for MVP: Genome wide association studies

Alternatively, the identification of frequent genetic variants showing higher allele frequencies in patients compared to controls allowed the identification of genes and biological pathways involved in MVP. Genome-wide association study analyses use frequent, genome-wide single-nucleotide polymorphisms (SNPs), and combine these results with information on expression data and epigenetic marks in biological tissues of interest, possibly involved in the disease.

The first genetic study by Dina et al. included 1,412 MVP cases and 2,439 controls, and identified six loci reaching genome-wide statistical significance, with a replication in 1,422 MVP cases and 6,779 controls (29). As also supported by functional analysis, this study demonstrated that two genes, *LMCD1* (LIM and cysteine-rich domains protein 1) and *TNS1* (tensin1), impair valve phenotype as confirmed in animal models (29). *TNS1* is localized in focal adhesions area that mediate cellular regulatory effects in response to ECM signals. *TNS1* KO mice present thickened posterior mitral leaflets, ECM defects and signs of myxomatous degeneration. Both *TNS1* and *LMCD1* knockdown (KD) zebrafish exhibit MV regurgitation. Also called Dyxin, *LMCD1* is part of the zinc finger proteins family, co-regulators of transcription. *LMCD1* has been exhibited as a repressor of GATA6 (GATA binding protein 6), an important regulator for cardiac development and activator of calcineurin/NFAT pathway also implied in cardiac development. Actually, both *TNS1* and *LMCD1* genes are involved in cellular proliferation and migration during valve development. Thus, it is plausible that variants in these genes are causing valvular defects through embryonic valvulogenesis defects.

**FIGURE 2:**
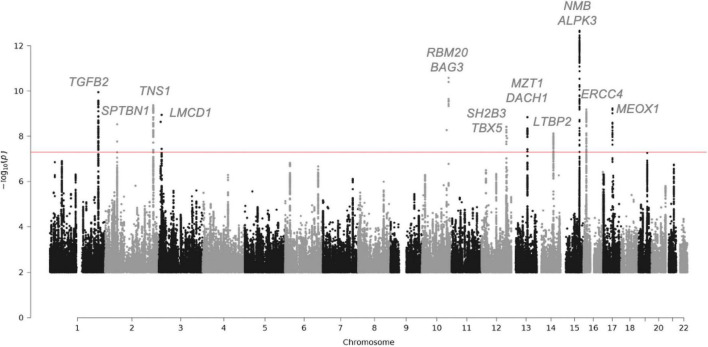
Manhattan plot showing the loci with genome-wide significance in the mitral valve prolapse meta-analysis and some candidate genes. Fourteen loci reached genome-wide significance (*P*-value, 5 × 10-8). Of the candidate genes from the meta-analysis, 15 genes are highlighted. The red line indicates the genome-wide significance cut-off. For the complete list of candidate genes, see Table 2.

This genetic analysis was greatly increased in the subsequent study by Roselli et al. that analyzed 4,884 patients with MVP and 434,649 controls (30) (Figure 2 and Table 2). In this study, data from the RoadMap epigenomics (31) and GTEx panel (32), as well as biochemical experiments on 111 cell lines and tissues were used. It allowed to catalog potential functional DNA regions, as well as the links between gene expression and genetic polymorphism. The power of this new study allowed the identification of 14 genetic loci pointing to new candidate genes associated with MVP, including non-ischemic cardiomyopathy genes. Although none of the main SNPs were in coding regions, for five hits there was at least one SNP in linkage disequilibrium with the lead SNPs located in an exon, thus pointing to five genes (*ALPK3*, *NMB*, *SH2B3*, *BAG3*, and *SMG6*) (30). Gene mapping includes the search for significantly associated SNPs, but also for multi-polymorphism associations including SNPs over a 10 Kb window surrounding and including the gene (MAGMA method) (33). Nine genes close to the GWAS top-hits showed an association with this method as well.

**TABLE 2:** Candidate genes from MVP meta-analysis and follow-up analysis.

	Chr	eGene	TWAS	NG	Proteomics	RNA-seq	MAGMA	Literature	Intronic	Missense
*TGFB2*	1			X	X	X	X			
*SPTBN1*	2	X		X	X	X	X		X	
*TNP1*	2			X						
*TNS1*								X		
*DIRC3*				X						
*LMCD1*	3	X		X	X	X		X	X	
*CAND2*	3			X	X	X				
*TMEM40*				X		X				
*RBM20*	10			X	X	X			X	
*BAG3*	10			X	X	X			X	X
*BRAP*	12				X	X				
*ATXN2*				X	X	X			X	
*SH2B3*						X				X
*ADAM1B*		X								
*TBX5*	12			X		X			X	
*MZT1*	13			X		X				
*DACH1*				X		X				
*LTBP2*	14				X	X				
*NMB*	15	X	X		X	X	X			X
*ALPK3*		X	X	X	X	X	X			X
*WDR73*		X	X			X	X			
*SEC11A*					X	X	X			
*ZNF592*				X			X			
*ZSCAN2*							X			
*UBE2Q2L*		X	X							
*CSPG4P12*		X								
*GOLGA2P7*		X								
*LIN00933*		X								
*RP11-182J1.14*		X								
*RP11-182J1.18*		X								
*ERCC4*	16			X		X				
*MEOX1*	17			X		X				
*GLIS1*	1			X			X		X	

eGene, expression quantitative trait locus gene; GWAS, genome-wide association study; MAGMA, multi-marker analysis of genomic annotation; MVP, mitral valve prolapse; NG, nearest gene(s); RNA-Seq, RNA sequencing; TWAS, transcriptome-wide association study. Each line corresponds to a candidate gene. Each column presents the lines of evidence for each gene. Cross indicate that the evidence is present.

In addition, the correlation between the presence of MVP and the expression levels of human genes, through a process of imputing the expression levels of all genes in patients and control data from a reference panel was tested. This tissue-specific TWAS (Transcriptomic Wide Association Study) (34) method was applied by imputing expression in cardiac ventricular and atrial tissue (from GTEx). This analysis allowed the *NMB* gene to be added to the list of genes showing simultaneous SNP and whole gene association.

The pathway analysis on genes near MVP-associated loci from the GWAS indicated that biological functions relevant to cell adhesion and migration during cardiac and valvular development are involved. The genes spanning biological mechanisms highly relevant to MVP included *GLIS1*, *TGFB2*, *ID2*, *TBX5*, *MSRA*, and *DMPK* (35). The transcription factor *GLIS1* has a potential role in mechanisms related to endothelial to mesenchymal transition and cell migration (36). In addition, in a meta-analysis with UK-Biobank MVP, Yu et al. replicated the association on *TNS1* and indicated that several gene sets enriched for MVP-associated genes are related to cardiac biology, such as cardiac ventricle formation, cardiac chamber formation and cardiac right ventricle morphogenesis (35).

In conclusion, the latest GWAS studies, combining genetic association, TWAS, in-house mRNA and protein expression, have confirmed *LMCD1* and identified *SPTBN1*, *LTBP2*, *TGFB2*, *NMB*, and *ALPK3* as potentially important in the occurrence of MVP. The complete list of candidate genes for the regions reaching GWAS significance are listed Table 2.

### Transcriptomics analyses

In addition to the above data, transcriptome analyses were performed in mitral valve leaflets from humans and animals with MVP. In humans, transcriptome analyses of mitral valve leaflets demonstrated changes in the expression of proteolytic genes, including metallothioneins, MMPs (matrix metalloproteinases), cathepsins, and ADAMTS (a disintegrin and metalloproteinase domain with thrombospondin), associated with enrichment of the TGF-beta pathway (37–39). Comparative gene expression profiling in the mitral valve leaflets of myxomatous MVP and FED MVP revealed distinct gene expression profiles, suggesting the involvement of different pathophysiological mechanisms. Downregulation of ADAMTS5 in myxomatous MVP has been associated with the accumulation of the versican proteoglycan in the leaflet extracellular matrix, contributing to leaflet remodeling (40). Interestingly, recent data in Cavalier King Charles Spaniel have identified gene expression changes in mitral leaflets associated with cardiomyocytes, coagulation and extra-cellular matrix remodeling (41). Comparative transcriptomic evaluation of acquired forms of MVP in humans and dogs revealed a common upregulated inflammatory response in diseased valves (42). In small-breed dogs, the major biological function altered involve inflammation, cell movement, cardiovascular development, extracellular matrix organization, and epithelial-to-mesenchymal transition (43). Finally, in the KI Flna-P637Q rat model, extracellular matrix organization, epithelial cell migration, response to mechanical stress, and immune cells were identified as the main signaling pathways leading to mitral valve myxomatous dystrophy (24). Hence, transcriptomic analyses performed in humans and animal models point toward common biological function and pathways.

## A genetic association between MVP and cardiomyopathy?

Myxomatous degeneration, also known as Barlow’s disease, associates bileaflet MVP, marked annular dilatation, frequent mitral annulus disjunction, and disproportionate left ventricular (LV) dilatation (44, 45) even in the absence of significant MR (46). Several hypotheses have been suggested to explain the intriguing disproportionate LV dilatation associated to myxomatous MVP, namely (1) an underestimation of volume overload linked to the pseudoaneurysmal prolapse sequestering a significant volume of blood (9, 47), (2) a compensatory mechanism to frequent ventricular ectopic beats (48), and (3) a unique type of associated cardiomyopathy (44) which could be linked to a genetic background. Indeed, histological examination of 68 cases of sudden death with MVP have shown unique features of cardiomyopathy including left ventricular fibrosis, associated hypertrophy, and degenerative features of the myocytes in 81% of cases (49).

Currently, there are little genetic data to establish a consistent link between MVP and a known cardiomyopathy phenotype. However, an association between *HCN4* (hyperpolarization-activated cyclic nucleotide channel 4) rare variants and MVP have been reported. Authors identified a complex pathologic association of sinus node dysfunction/bradycardia, ventricular and atrial arrhythmias, LV non-compaction, and MVP (50, 51). Of note, *HCN4* intervenes in myocardium and conduction system development. This association suggests that common genetic and molecular mechanisms can lead to rhythm/conduction, myocardial, and MV defects, reinforcing the hypothesis of a potential molecular interplay in myocardial and MV development. Mutations in the *DCHS1* gene, encoding a member of the cadherin superfamily involved in cell-cell adhesion molecules, have been identified as causing myxomatous MVP (26). Interestingly, cadherins are important components of the structural and functional cohesion of cardiomyocytes at the level of desmosomes, also known as maculae adherents. Desmosomes are one of the strongest types of cell-cell adhesion found in tissues submitted to intense mechanical stress. In addition, genetic abnormalities of some cadherins involved in cardiomyocyte desmosomes lead to a unique type of cardiomyopathy, the arrhythmogenic cardiomyopathy, which sometimes takes the form of an isolated dilated cardiomyopathy (52). In a recent study, out of 101 patients with MVP predominantly of Barlow type who underwent extensive gene testing, eight patients (8%) had a likely pathogenic variant in four cardiomyopathy genes (*DSP*, *HCN4*, *MYH6*, and *TTN*), in favor of a common genetic origin for both myocardial and MV disease (53) but we cannot rule out a chance association. It is worth noting that *TTN* encodes for the giant sarcomeric protein titin and is regarded the cause of ≈25% of familial dilated cardiomyopathy. Finally, one can hypothesize that a cardiomyopathy gene defect could worsens LV remodeling related to Barlow MVP and MR volume overload.

As already discussed, the most recent meta-analysis identified new candidate genes associated with MVP, including cardiomyopathy genes such as *ALPK3*, *BAG3*, and *RBM20* (30). In a sub-analysis of the association of genetic architecture and cardiovascular characteristics, the authors observed a strong genetic correlation between MVP and larger ventricular volumes, and a trend toward a positive correlation between MVP and non-ischemic cardiomyopathy. The observed association of MVP to LV dilatation is likely due to chronic MR, but could also be due in part to a common genetic defect of dilated cardiomyopathy and MVP. Whereas mutations in *ALPK3* have been associated with non-sarcomeric hypertrophic cardiomyopathy or dilated cardiomyopathy, *BAG3* and *RBM20* genes have been associated with dilated cardiomyopathy phenotype further highlighting a potential link between MVP and cardiomyopathy (Figure 2 and Table 2).

## Arrhythmogenic MVP

Most sudden deaths in patients with MVP occur in the presence of severe MR, corresponding to a decompensated stage of LV remodeling related to volume overload (54, 55). Arrhythmic MVP (46, 56), which at worst results in polymorphic ventricular arrhythmia that can eventually lead to sudden death (malignant MVP), has long been recognized a rare complication of myxomatous MVP (57) (Figure 1D). High-risk features include bileaflet MVP, mitral annulus disjunction (a classical finding in myxomatous bileaflet MVP), ST-T wave abnormalities in inferior or lateral leads, frequent complex ventricular ectopy arising from the MV apparatus, and finally LV focal myocardial fibrosis (44, 55, 57). However, the most classic form of arrhythmic MVP manifests as frequent, monomorphic ventricular ectopy, usually originating from the MV apparatus and in particular from the papillary muscles (58, 59). This classical form is generally either asymptomatic or symptoms are limited to isolated palpitations, but all situations exist between this benign form and malignant MVP. Owing to the low but significant increase risk of sudden death in MVP, recent consensus recommendations suggest to change the paradigm of the clinical work-up of MVP and to include a systematic 24-h ECG recording to unmask ventricular arrhythmia (46). Exercise test can also unmask ventricular arrhythmia not evidenced at rest (59). Several intricate factors are probably at the origin of ventricular arrhythmia in MVP, namely (1) abnormal myocardial stress during systole related to MV apparatus abnormalities, (2) myocardial remodeling with areas of myocardial fibrosis possibly favored by an underlying cardiomyopathy or by abnormal systolic stress, (3) electrical abnormalities of the myocardial contractile and conductive tissue, and (4) genetic factors. Our current knowledge of genetic factors favoring the occurrence of ventricular arrhythmia in patients with MVP remains limited. Only few publications have reported a potential association of MVP and pathogenic variants of the *FLNC* (60) or *LMNA* (61) genes in patients with ventricular arrhythmia. However, in a recent whole exome molecular autopsy in unexplained sudden death in young patients, MVP prevalence (7.8%) was higher than expected in the general population and pathogenic/likely pathogenic variants in cardiomyopathy-and channelopathy-susceptibility genes were over-represented (62). The frequency of MVP in the general population necessarily leads to incidental association between an inherited arrhythmia disease or a genetic variant of cardiomyopathy and MVP (63), although these factors might be synergistically associated according to the double hit theory (61, 62) and could increase the risk of sudden death. The genetic background of ventricular arrhythmia in MVP will require further investigations.

## Animal models of non-syndromic MVP

In genetic diseases, animal models are essential, (1) to confirm the link between the genetic anomaly and the pathology, (2) to characterize the functional and structural organs anomalies, (3) to identify the impacted molecular pathways, and finally, (4) to test therapeutics that could slow down or stop the evolution of the disease.

**FIGURE 3:**
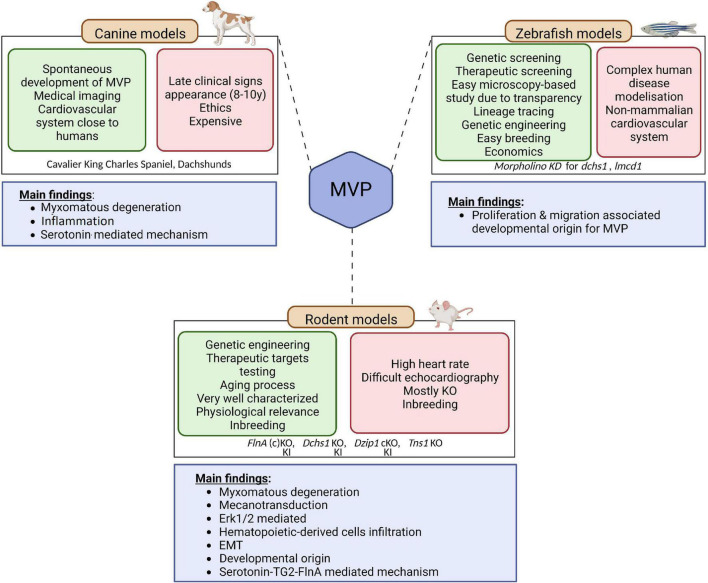
Animal models of MVP. Three main categories of animal models are currently available to study MVP, the canine model, the rodent model, and the zebrafish model. The advantages of each are presented in the green squares and downsides are presented in the red squares. The main finding coming out of the models are depicted in the blue square underneath. EMT, endothelial to mesenchymal transition; KO, knock-out; KI, knock-in; MVP, mitral valve prolapse; SERT, serotonin transporter; TG2, transglutaminase 2. Created with BioRender.com.

In MVP, three main categories of animal models are currently available, the canine model, the rodent model, and the zebrafish model (Figure 3). It should be noted that the contribution of the three main animal models to the improvement of knowledge regarding humans MVP is different. Although MVP in the small-breed dog may be considered to have greater histological and clinical proximity to human MVP, the rodent and zebrafish models are of primary interest for translational research because they can be easily manipulated to express a genetic defect identified in humans.

Small-breed dogs, such as Cavalier King Charles Spaniel and Dachshunds, are well known to spontaneously develop myxomatous MVP. Interestingly, this physiologically relevant large mammalian animal model develops the three-layered valves and the MVP phenotype observed in these animals are close to what is described in humans, making this intermediary translational model of interest to study mechanisms associated with the disease (64). Indeed, on histology, MVP leaflet tissue shows thickening of the spongiosa layer, altered collagen, and glycosaminoglycan infiltration, in agreement with the Barlow disease in humans (65). As in humans, MVP is considered transmissible in small-breed dogs (66). Hence small-breed dogs could represent a resource for uncovering the genetic basis of MVP (65), even though first attempts based on whole genome sequencing did not identify genetic cause in the homologous genes involved in the human disease (66). The analyses performed on canine model of MVP suggested that MVP would be linked to cell structure and ECM remodeling, immunity and inflammation as well as high serum serotonin, 5-HT2B receptor overexpression and serotonin transporter SERT downregulation (41, 67–69). However, from a research standpoint, the late and very high incidence of MVP in these dogs, with the almost constant presence of MVP in old small-breed dogs such as Cavalier King Charles Spaniels (69), the important karyotype differences compared to humans (69), as well as ethical concern regarding the use of dogs for research, considerably limit their usefulness to study genetics and molecular mechanisms.

Researchers thus turned toward rodent-based animal models (70),^1^ the most commonly and readily engineered models used in laboratories. Even if the mitral valve of rodents does not present clear layer-structure as observed in humans and dogs, but a polarized leaflet structure with collagen strip and proteoglycan content along the leaflets, rodent models offer important benefit for research and open the way to decipher the molecular mechanisms behind the development of the pathology. As discussed previously, genetic studies performed in the last decades unraveled the association of several genes with MVP. The first causal gene, the *FLNA* gene that encodes for Filamin A (FlnA) protein, was identified in 2007 (23). Analysis of the *FlnA* KO mice model have reinforced the genetic findings relating FlnA and MVP: Feng et al. have reported significant outflow tract defects in *FlnA* KO embryos, thus suggesting important impact of FlnA in the development of MV (71). However, the developmental lethality of this model precluded any clear identification of the mechanisms behind the FlnA/MVP association. To overpass this limitation, Sauls et al. generated a conditional KO mice, where FlnA was specifically deleted in endothelial-derived cells using Tie2/Cre method (25). They showed that adult valve degeneration originates from developmental defects where they reported fetal valve extracellular matrix (ECM) remodeling. They showed an increased infiltration of extra cardiac cells, such as bone marrow-derived cells (see dedicated section below), and/or an activation of the Erk pathway, suggesting that immune cells and the regulation of Erk pathway could represent a potential therapeutic option for MVP patients (72). However, the complete lack of a hub protein like FlnA, involved in many signaling networks, can hardly recapitulate the effects of a single MVP-associated point mutation and thus considerably limits the ability to unravel fine pathophysiological mechanisms leading to MVP. Fortunately, a knock-In (KI) FlnA-P637Q rat model carrying the *FLNA* variant identified in a large human family (1, 2, 22) has been recently generated and phenotyped (24, 73). This animal model mimics human myxomatous MVP, offering a unique opportunity to decipher the pathophysiological mechanisms related to this disease. ECM remodeling, epithelial cell migration, response to mechanical stress and the contribution of immune cells were highlighted as the main pathological pathways leading to *FLNA* myxomatous MVP (24). The importance of immune cells in the development of MVP is a recent concept that may offer new therapeutic perspectives in MVP (42).

Relying on other causal genes that have been identified (*DCHS1*, polymorphisms in the region of *LMCD1* and *TNS1*) several other models were generated. Knockdown zebrafish suggested that cellular proliferation and migration were the main processes involved in the development of MVP. Although zebrafish models present several advantages, such as microscopy-based study and easy lineage tracing (74), its physiological relevance remains limited to study complex pathophysiology occurring in human disease. Therefore, genetically engineered mice have been generated. *Dchs1* KO mice developed thickened mitral leaflet and MVP with altered cellular patterning (26), *Tns1* KO mice exhibited enlarged posterior MV leaflet with evidence of myxomatous degeneration, as indicated by increased proteoglycan content and loss of normal matrix stratification (29). More recently, a cilia gene, *DZIP1*, has been identified in MVP families (27). Thanks to the generation and study of a KI and conditional KO mice model, Toomer et al. demonstrated the pathogenicity of *DZIP1* specific mutation highlighting a role for primary cilia in the development of myxomatous MVP (27).

The zebrafish model has a special place in the arsenal of animal models (Figure 3). The zebrafish model is used to characterize human pathologies and, in MVP, to validate the association between a genetic defect and the development of mitral valve dysfunction (29, 36). However, this model is limited by the characteristics of the fish heart and its extremely small size. The interest of this model in the future, beyond the genetic aspect, lies in the possibility of rapidly testing the effect of numerous drugs that could prevent or slow the development of MVP.

Overall, canine models as well as rodent-based models for non-syndromic MVP suggest that common causal pathways, such as endothelial to mesenchymal transition (EMT), mechano-transduction, and immune cells, are involved in the development of MVP.

## Pathophysiological pathways of MVP

**FIGURE 4:**
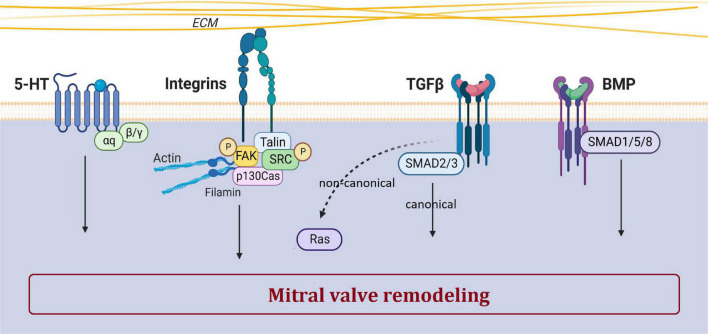
Main biological pathways involved in MVP. This figure shows the involvement of serotonin, integrin, TGFβ, and BMP pathways in the pathogenesis of MVP. The dotted arrow corresponds to the non-canonical TGFβ pathway. Created with Biorender.com.

Rather than ECM remodeling, mechano-transduction, EMT, immune cells and ciliation alterations have been identified as important mechanisms leading to the development of MVP. The main biological pathways involved in MVP development and dysfunction includes integrins, the TGFβ pathway, and supposedly serotonin (Figure 4). Mechanisms and biological pathways involved in MVP development are likely also involved in the development of other heart valve diseases such as tricuspid valve prolapse.

### Transforming growth factor β (TGFβ)

TGFβ superfamily, a large family of regulatory proteins, regulates many cellular mechanisms and is a major player in valvulogenesis and valve pathophysiology, involved in EMT but also in valve remodeling (26). Nevertheless, the precise mechanisms by which TGFβ participates in MVP are still poorly understood.

The study of fibrillin-1 mutations associated with Marfan syndrome has highlighted the prominent role of the TGFβ pathway in MVP. Indeed, excessive signaling of TGFβ superfamily members has been evidenced in human, canine and murine tissues of Marfan syndrome MVP (2, 41). Transcriptomic studies reported increased gene expression of TGFβ2 in human dystrophic MV compared with control MV (64). Furthermore, various KO mice models for Smad isoforms develop cardiac dysfunction and defects in valvulogenesis (75). Lastly, in the multi-omics analysis (i.e., RNA-seq and ATAC-seq) we performed in our unique animal model of MVP, the FlnA-P637Q KI rat, TGFβ pathway was highlighted as one of the main player in the MVP pathogenesis (24).

### Serotonin

The serotonin (5-hydroxytryptamine or 5-HT) pathway plays a major role in cardiovascular pathophysiology, participates in valvulogenesis and valve remodeling (64, 68). Serotonin receptor (5-HTR) agonist drugs, such as benfluorex (Mediator^®^), stimulate serotonergic pathways leading to valve degeneration with increased VICs proliferation, endocardial damage and ECM accumulation (76).

Canine models of spontaneous MVP, such as the Cavalier Kings Charles, have demonstrated increased abundance of THP1, 5-HT2BR, a decrease in SERT, and an increase in circulating 5-HT levels in platelets and mitral leaflets (64, 68). Serotonylation by TG2 is involved in matrix remodeling, and thus in MVP. Interestingly, in KO mice developing MVP, maturation and matrix remodeling during embryogenesis have been shown as dependent on 5-HT/TG2/FlnA interactions (25). Lastly, the FlnA rat model presented doubled *Htr2a* transcript expression in myxomatous MVP, suggesting once again the involvement of serotonin pathway in MVP (24). However, the role of serotonin in the development or progression of MVP remains speculative, so further studies are needed to confirm the potential link between serotonin-related mechanisms and MVP.

### Ciliation

Cilia are structures formed by microtubules that extend from the cytoplasm to the outside of the cell. Primary cilia transmit mechanical, electrical and chemical signals depending on the spatio-temporal context. In this way, they coordinate signals from various signaling pathways to influence cell survival and differentiation as well as tissue organization (77).

In recent years, primary cilia have been recognized to play a central role in the biomechanics and molecular regulation of cardiac development (77), but the involved mechanisms remain poorly understood. Primary cilia are present on the surface of interstitial cells of the developing outflow tract cushion at embryonic stages E11.5 and E13.5, but are rarely seen on the valvular endocardium (27). Although still debated, primary cilia could regulate the production of different components of ECM.

A recently developed mouse model with the rare mutation in *DZIP1* confirmed the involvement of cilia in the development of myxomatous MVP (27). In addition, immunohistological analysis of *Dchs1* KO and *FlnA* KO mice also showed a significant reduction in the length of primary MV cells cilia (27). Recent data suggest that the alteration of primary cilia mechanosensing function is involved in profibrotic pathways, favoring LV fibrosis in mice and humans with MVP (78). We also recently highlighted the implication of cilia in the FlnA KI rat model developing myxomatous MVP (24).

### Endothelial-to-mesenchymal transition (EMT)

Happening at embryonic stage E9.5 in mice, EMT relates to the transition of endocardial cells to mesenchymal cells, and their simultaneous migration into the cardiac jelly during development to subsequently remodel ECM and give rise to valvular structure (79–82).

The EMT is mediated by TGFβ superfamily members (i.e., TGF-β/Smad, Notch, Wnt/β-catenin, VEGF). The entailment of EMT in MVP remains questioning and controversial as its signature in pathologic valves is not clearly identified. Although, *in vitro* and canine as well as sheep histologic studies reported EMT in MVP (43, 83–86), neither mice histologic studies using CD31 and α-SMA double-positive cells as an indicator of EMT, nor the analysis of EMT transcription factors, reported presence of EMT (87). In addition, *in vivo* transformation of valvular endothelial cells (VECs) into VICs has never been reported (87). Whereas lineage tracing allowed to characterize adult VICs origin, no evidence of EMT during adult valve homeostasis or valve pathogenesis was observed and further studies are needed to understand the potential role of this mechanisms in the development of the disease.

### Immune cells

By contrast, lineage tracing revealed the recruitment of bone-marrow derived cells both in healthy and myxomatous MV. Monocytes are established actors of tissue homeostasis, wound repair and disease. Although MVP was considered as non-inflammatory disease for a long time (88), CD45 + hematopoietic cells have been reported in human, sheep (89, 90) and murine (42, 72, 87, 91) myxomatous MVP. Most recent study by Kim et al. demonstrated the dominant role of monocyte-derived macrophages and the associated pro-inflammatory environment in the development of myxomatous MV dystrophy (42, 87) in a mouse model of Marfan syndrome. Furthermore, exacerbated inflammatory response was observed in dystrophic MV of human, dogs and mice, suggesting immune activity involvement in the progression of the disease. We also recently observed an enrichment in pathways related to chemotaxis and immune cell migration in the myxomatous mitral leaflets of the FlnA KI rat model, reinforcing the evidence of crucial role of immune cells in the pathophysiological mechanisms leading to myxomatous MVP remodeling (24).

### Mechanobiology

The MV is submitted to different mechanical forces throughout life since its development, during maturation, and after birth. VECs and VICs, especially of aortic and MV, have been shown to adapt to mechanical stress conditions (92, 93).

Mechanical stimuli are sensed through sensory proteins and mechanoreceptors such as mechanosensitive ion channels, annexin V and integrins (Figure 4) (94). Cyclic stretching induces a myofibroblastic phenotype with an increased expression of TGFβ2, and an activation of small protein RhoGTPases and the MEK/ERK pathway, while Smad2/3 effectors of the TGFβ pathway does not respond to stress (95).

To conclude, the pathophysiological mechanisms and molecular pathways involved in the development and progression of MVP are being deciphered, involving the TGF-β pathway, serotonin pathway, with disturbances in mechano-transduction, ciliation, possibly EMT, and immune cell-related valve remodeling.

## Familial screening and genetic counseling

The inheritance of MVP raises the question of systematic screening at least of first-degree relatives of probands with severe MVP, especially if there is a family history of surgical MVP or life-threatening arrhythmic MVP. Familial screening is a common practice to identify individuals at risk for some genetic condition but has not yet been clearly recommended in guidelines for MVP (46). Clinical interrogation of family history can identify families with severe, recurrent MVP or MR (13) and lead to ECG and echocardiographic screening of first-degree relatives, which may allow preclinical diagnosis and better follow-up. Although the lifetime rate of MV surgery is relatively low in the overall MVP population, estimated at approximately 6–7% at age 75 years (96), it may be as high as 70% in men at age 70 years in Filamin A-MVP (2), which should be incentive to familial screening. Screening for MVP by echocardiography in selected individuals is a simple procedure that will allow appropriate follow-up and timely intervention. In clinical practice, familial screening could be proposed to first-degree relatives in case of severe or recurrent form of MVP in the family.

Finally, the value of genetic counseling remains limited in MVP because of limited genetic data. However, a rapid clinical questioning and examination looking for the main syndromic features can reveal syndromic signs of Marfan syndrome or MASS (mitral aorta skeleton and skin phenotype) that would benefit from genetic diagnosis and counseling. As a reminder, the first sign of Marfan syndrome was MVP in 12% of a large series of Marfan syndrome (19).

## Author contributions

CoD, RC, JM, J-JS, ChD, and TL performed the data search and drafted the manuscript. RC, SL, NB-N, MY, OH, CS-S, J-MS, NP, and TL critically revised the draft. All authors contributed to the conception of this work and approved the final version of the manuscript.

## Footnotes

1
http://www.mousemine.org/mousemine/begin.do

## References

[1] Levine R Hagége A Judge D Padala M Dal-Bianco J Aikawa E Mitral valve disease–morphology and mechanisms. *Nat Rev Cardiol.* (2015) 12:689–710. 10.1038/nrcardio.2015.161 26483167 PMC4804623

[2] Le Tourneau T Le Scouarnec S Cueff C Bernstein D Aalberts J Lecointe S New insights into mitral valve dystrophy: a Filamin-A genotype-phenotype and outcome study. *Eur Heart J.* (2018) 39:1269–77. 10.1093/eurheartj/ehx505 29020406 PMC5905589

[3] Le Tourneau T Mérot J Rimbert A Le Scouarnec S Probst V Le Marec H Genetics of syndromic and non-syndromic mitral valve prolapse. *Heart.* (2018) 104:978–84. 10.1136/heartjnl-2017-312420 29352010 PMC6168077

[4] Adams D Rosenhek R Falk V. Degenerative mitral valve regurgitation: best practice revolution. *Eur Heart J.* (2010) 31:1958–66. 10.1093/eurheartj/ehq222 20624767 PMC2921508

[5] Fornes P Heudes D Fuzellier J Tixier D Bruneval P Carpentier A. Correlation between clinical and histologic patterns of degenerative mitral valve insufficiency: a histomorphometric study of 130 excised segments. *Cardiovasc Pathol.* (1999) 8:81–92. 10.1016/s1054-880700021-0 10724505

[6] Delling F Rong J Larson M Lehman B Osypiuk E Stantchev P Familial clustering of mitral valve prolapse in the community. *Circulation.* (2015) 131:263–8. 10.1161/CIRCULATIONAHA.114.012594 25361552 PMC4301989

[7] Nesta F Leyne M Yosefy C Simpson C Dai D Marshall J New locus for autosomal dominant mitral valve prolapse on chromosome 13: clinical insights from genetic studies. *Circulation.* (2005) 112:2022–30. 10.1161/CIRCULATIONAHA.104.516930 16172273

[8] Delling F Rong J Larson M Lehman B Fuller D Osypiuk E Evolution of mitral valve prolapse: insights from the Framingham Heart Study. *Circulation.* (2016) 133:1688–95. 10.1161/CIRCULATIONAHA.115.020621 27006478 PMC4856536

[9] Barlow J Bosman C. Aneurysmal protrusion of the posterior leaflet of the mitral valve. An auscultatory-electrocardiographic syndrome. *Am Heart J.* (1966) 71:166–78. 10.1016/0002-870390179-7 4159172

[10] Monteleone P Fagan L. Possible X-linked congenital heart disease. *Circulation.* (1969) 39:611–4. 10.1161/01.cir.39.5.611 5787314

[11] Devereux R Brown W Kramer-Fox R Sachs I. Inheritance of mitral valve prolapse: effect of age and sex on gene expression. *Ann Intern Med.* (1982) 97:826–32. 10.7326/0003-4819-97-6-826 7149490

[12] Delling F Li X Li S Yang Q Xanthakis V Martinsson A Heritability of mitral regurgitation: observations from the Framingham Heart Study and Swedish population. *Circ Cardiovasc Genet.* (2017) 10:e001736. 10.1161/CIRCGENETICS.117.001736 28993406 PMC5679298

[13] Hiemstra Y Wijngaarden A Bos M Schalij M Klautz R Bax J Familial occurrence of mitral regurgitation in patients with mitral valve prolapse undergoing mitral valve surgery. *Eur J Prev Cardiol.* (2020) 27:272–80. 10.1177/2047487319874148 31475862 PMC7008556

[14] Kyndt F Schott J Trochu J Baranger F Herbert O Scott V Mapping of X-linked myxomatous valvular dystrophy to chromosome Xq28. *Am J Hum Genet.* (1998) 62:627–32. 10.1086/301747 9497244 PMC1376942

[15] Disse S Abergel E Berrebi A Houot A Le Heuzey J Diebold B Mapping of a first locus for autosomal dominant myxomatous mitral-valve prolapse to chromosome 16p11.2-p12.1. *Am J Hum Genet.* (1999) 65:1242–51. 10.1086/302624 10521289 PMC1288276

[16] Freed L Acierno J Dai D Leyne M Marshall J Nesta F A locus for autosomal dominant mitral valve prolapse on chromosome 11p15.4. *Am J Hum Genet.* (2003) 72:1551–9. 10.1086/375452 12707861 PMC1180315

[17] Morningstar J Nieman A Wang C Beck T Harvey A Norris R. Mitral valve prolapse and its motley crew-syndromic prevalence, pathophysiology, and progression of a common heart condition. *J Am Heart Assoc.* (2021) 10:e020919. 10.1161/JAHA.121.020919 34155898 PMC8403286

[18] Loeys B Dietz H Braverman A Callewaert B De Backer J Devereux R The revised Ghent nosology for the Marfan syndrome. *J Med Genet.* (2010) 47:476–85. 10.1136/jmg.2009.072785 20591885

[19] Attias D Stheneur C Roy C Collod-Béroud G Detaint D Faivre L Comparison of clinical presentations and outcomes between patients with TGFBR2 and FBN1 mutations in Marfan syndrome and related disorders. *Circulation.* (2009) 120:2541–9. 10.1161/CIRCULATIONAHA.109.887042 19996017

[20] Détaint D Faivre L Collod-Beroud G Child A Loeys B Binquet C Cardiovascular manifestations in men and women carrying a FBN1 mutation. *Eur Heart J.* (2010) 31:2223–9. 10.1093/eurheartj/ehq258 20709720

[21] Gouda P Kay R Habib M Aziz A Aziza E Welsh R. Clinical features and complications of Loeys-Dietz syndrome: a systematic review. *Int J Cardiol.* (2022) 362:158–67. 10.1016/j.ijcard.2022.05.065 35662564

[22] Trochu J Kyndt F Schott J Gueffet J Probst V Bénichou B Clinical characteristics of a familial inherited myxomatous valvular dystrophy mapped to Xq28. *J Am Coll Cardiol.* (2000) 35:1890–7. 10.1016/s0735-109700617-3 10841240

[23] Kyndt F Gueffet J Probst V Jaafar P Legendre A Le Bouffant F Mutations in the gene encoding Filamin A as a cause for familial cardiac valvular dystrophy. *Circulation.* (2007) 115:40–9. 10.1161/CIRCULATIONAHA.106.622621 17190868

[24] Delwarde C Toquet C Aumond P Kayvanjoo A Foucal A Le Vely B Multimodality imaging and transcriptomics to phenotype mitral valve dystrophy in a unique knock-in Filamin-A rat model. *Cardiovasc Res.* (2022). 10.1093/cvr/cvac136 [Online ahead of print]. 36001550

[25] Sauls K de Vlaming A Harris B Williams K Wessels A Levine R Developmental basis for Filamin-A-associated myxomatous mitral valve disease. *Cardiovasc Res.* (2012) 96:109–19. 10.1093/cvr/cvs238 22843703 PMC3444235

[26] Durst R Sauls K Peal D deVlaming A Toomer K Leyne M Mutations in DCHS1 cause mitral valve prolapse. *Nature.* (2015) 525:109–13. 10.1038/nature14670 26258302 PMC4720389

[27] Toomer K Yu M Fulmer D Guo L Moore K Moore R Primary cilia defects causing mitral valve prolapse. *Sci Transl Med.* (2019) 11:eaax0290. 10.1126/scitranslmed.aax0290 31118289 PMC7331025

[28] Guo L Beck T Fulmer D Ramos-Ortiz S Glover J Wang C DZIP1 regulates mammalian cardiac valve development through a Cby1-β-catenin mechanism. *Dev Dyn.* (2021) 250:1432–49. 10.1002/dvdy.342 33811421 PMC8518365

[29] Dina C Bouatia-Naji N Tucker N Delling F Toomer K Durst R Genetic association analyses highlight biological pathways underlying mitral valve prolapse. *Nat Genet.* (2015) 47:1206–11. 10.1038/ng.3383 26301497 PMC4773907

[30] Roselli C Yu M Nauffal V Georges A Yang Q Love K Genome-wide association study reveals novel genetic loci: a new polygenic risk score for mitral valve prolapse. *Eur Heart J.* (2022) 43:1668–80. 10.1093/eurheartj/ehac049 35245370 PMC9649914

[31] Roadmap Epigenomics Consortium Kundaje A Meuleman W Ernst J Bilenky M Yen A Integrative analysis of 111 reference human epigenomes. *Nature.* (2015) 518:317–30. 10.1038/nature14248 25693563 PMC4530010

[32] GTEx Consortium. The genotype-tissue expression (GTEx) project. *Nat Genet.* (2013) 45:580–5. 10.1038/ng.2653 23715323 PMC4010069

[33] de Leeuw C Mooij J Heskes T Posthuma D. MAGMA: generalized gene-set analysis of GWAS data. *PLoS Comput Biol.* (2015) 11:e1004219. 10.1371/journal.pcbi.1004219 25885710 PMC4401657

[34] Gusev A Ko A Shi H Bhatia G Chung W Penninx B Integrative approaches for large-scale transcriptome-wide association studies. *Nat Genet.* (2016) 48:245–52. 10.1038/ng.3506 26854917 PMC4767558

[35] Yu M Kyryachenko S Debette S Amouyel P Schott J Le Tourneau T Genome-wide association meta-analysis supports genes involved in valve and cardiac development to associate with mitral valve prolapse. *Circ Genom Precis Med.* (2021) 14:e003148. 10.1161/CIRCGEN.120.003148 34461747 PMC8530910

[36] Yu M Georges A Tucker N Kyryachenko S Toomer K Schott J Genome-wide association study-driven gene-set analyses, genetic, and functional follow-up suggest GLIS1 as a susceptibility gene for mitral valve prolapse. *Circ Genom Precis Med.* (2019) 12:e002497. 10.1161/CIRCGEN.119.002497 31112420 PMC6532425

[37] Greenhouse D Murphy A Mignatti P Zavadil J Galloway A Balsam L. Mitral valve prolapse is associated with altered extracellular matrix gene expression patterns. *Gene.* (2016) 586:56–61. 10.1016/j.gene.2016.04.004 27063507

[38] Hulin A Deroanne C Lambert C Dumont B Castronovo V Defraigne J Metallothionein-dependent up-regulation of TGF-β2 participates in the remodelling of the myxomatous mitral valve. *Cardiovasc Res.* (2012) 93:480–9. 10.1093/cvr/cvr337 22180604

[39] Sainger R Grau J Branchetti E Poggio P Seefried W Field B Human myxomatous mitral valve prolapse: role of bone morphogenetic protein 4 in valvular interstitial cell activation. *J Cell Physiol.* (2012) 227:2595–604. 10.1002/jcp.22999 22105615 PMC3288540

[40] Absi T Galindo C Gumina R Atkinson J Guo Y Tomasek K Altered ADAMTS5 expression and versican proteolysis: a possible molecular mechanism in Barlow’s disease. *Ann Thorac Surg.* (2018) 105:1144–51. 10.1016/j.athoracsur.2017.11.035 29248417

[41] Markby G Macrae V Corcoran B Summers K. Comparative transcriptomic profiling of myxomatous mitral valve disease in the cavalier King Charles spaniel. *BMC Vet Res.* (2020) 16:350. 10.1186/s12917-020-02542-w 32967675 PMC7509937

[42] Kim A Xu N Umeyama K Hulin A Ponny S Vagnozzi R Deficiency of circulating monocytes ameliorates the progression of myxomatous valve degeneration in Marfan syndrome. *Circulation.* (2020) 141:132–46. 10.1161/CIRCULATIONAHA.119.042391 31928435 PMC7017394

[43] Lu C Liu M Culshaw G Clinton M Argyle D Corcoran B. Gene network and canonical pathway analysis in canine myxomatous mitral valve disease: a microarray study. *Vet J.* (2015) 204:23–31. 10.1016/j.tvjl.2015.02.021 25841900

[44] Constant Dit Beaufils A Huttin O Jobbe-Duval A Senage T Filippetti L Piriou N Replacement myocardial fibrosis in patients with mitral valve prolapse: relation to mitral regurgitation, ventricular remodeling, and arrhythmia. *Circulation.* (2021) 143:1763–74. 10.1161/CIRCULATIONAHA.120.050214 33706538

[45] Pype L Bertrand P Paelinck B Heidbuchel H Van Craenenbroeck E Van De Heyning C. Left ventricular remodeling in non-syndromic mitral valve prolapse: volume overload or concomitant cardiomyopathy? *Front Cardiovasc Med.* (2022) 9:862044. 10.3389/fcvm.2022.862044 35498019 PMC9039519

[46] Sabbag A Essayagh B Barrera J Basso C Berni A Cosyns B EHRA expert consensus statement on arrhythmic mitral valve prolapse and mitral annular disjunction complex in collaboration with the ESC Council on valvular heart disease and the European Association of Cardiovascular Imaging endorsed cby the Heart Rhythm Society, by the Asia Pacific Heart Rhythm Society, and by the Latin American Heart Rhythm Society. *Europace.* (2022) 24:1981–2003. 10.1093/europace/euac125 35951656 PMC11636573

[47] El-Tallawi K Kitkungvan D Xu J Cristini V Yang E Quinones M Resolving the disproportionate left ventricular enlargement in mitral valve prolapse due to Barlow disease: insights from cardiovascular magnetic resonance. *JACC Cardiovasc Imaging.* (2021) 14:573–84. 10.1016/j.jcmg.2020.08.029 33129724

[48] Basso C Perazzolo Marra M Rizzo S De Lazzari M Giorgi B Cipriani A Arrhythmic mitral valve prolapse and sudden cardiac death. *Circulation.* (2015) 132:556–66. 10.1161/CIRCULATIONAHA.115.016291 26160859

[49] Garbi M Lancellotti P Sheppard M. Mitral valve and left ventricular features in malignant mitral valve prolapse. *Open Heart.* (2018) 5:e000925. 10.1136/openhrt-2018-000925 30364469 PMC6196952

[50] Milano A Vermeer A Lodder E Barc J Verkerk A Postma A HCN4 mutations in multiple families with bradycardia and left ventricular noncompaction cardiomyopathy. *J Am Coll Cardiol.* (2014) 64:745–56. 10.1016/j.jacc.2014.05.045 25145517

[51] Schweizer P Koenen M Katus H Thomas D. A distinct cardiomyopathy: HCN4 syndrome comprising myocardial noncompaction, bradycardia, mitral valve defects, and aortic dilation. *J Am Coll Cardiol.* (2017) 69:1209–10. 10.1016/j.jacc.2016.10.085 28254188

[52] Piriou N Marteau L Kyndt F Serfaty J Toquet C Le Gloan L Familial screening in case of acute myocarditis reveals inherited arrhythmogenic left ventricular cardiomyopathies. *ESC Heart Fail.* (2020) 7:1520–33. 10.1002/ehf2.12686 32356610 PMC7373927

[53] van Wijngaarden A Hiemstra Y Koopmann T Ruivenkamp C Aten E Schalij M Identification of known and unknown genes associated with mitral valve prolapse using an exome slice methodology. *J Med Genet.* (2020) 57:843–50. 10.1136/jmedgenet-2019-106715 32277046

[54] Grigioni F Enriquez-Sarano M Ling L Bailey K Seward J Tajik A Sudden death in mitral regurgitation due to flail leaflet. *J Am Coll Cardiol.* (1999) 34:2078–85. 10.1016/s0735-109700474-x 10588227

[55] Basso C Iliceto S Thiene G Perazzolo Marra M. Mitral valve prolapse, ventricular arrhythmias, and sudden death. *Circulation.* (2019) 140:952–64. 10.1161/CIRCULATIONAHA.118.034075 31498700

[56] Essayagh B Sabbag A Antoine C Benfari G Yang L Maalouf J Presentation and outcome of arrhythmic mitral valve prolapse. *J Am Coll Cardiol.* (2020) 76:637–49. 10.1016/j.jacc.2020.06.029 32762897

[57] Pocock W Bosman C Chesler E Barlow J Edwards J. Sudden death in primary mitral valve prolapse. *Am Heart J.* (1984) 107:378–82. 10.1016/0002-870390389-2 6695671

[58] Hong-TaoYuan N Yang M Zhong L Lee Y Vaidya V Asirvatham S Ventricular premature contraction associated with mitral valve prolapse. *Int J Cardiol.* (2016) 221:1144–9. 10.1016/j.ijcard.2016.06.252 27522301

[59] Guenancia C Pace N Hossu G Selton-Suty C Mandry D Beaumont M Prevalence and determinants of PVCs originating from the mitral apparatus in patients with MVP. *JACC Clin Electrophysiol.* (2022) 8:526–8. 10.1016/j.jacep.2021.12.005 35450608

[60] Bains S Tester D Asirvatham S Noseworthy P Ackerman M Giudicessi J. A Novel truncating variant in FLNC-encoded Filamin C may serve as a proarrhythmic genetic substrate for arrhythmogenic bileaflet mitral valve prolapse syndrome. *Mayo Clin Proc.* (2019) 94:906–13. 10.1016/j.mayocp.2018.11.028 30935706

[61] Appignani M Khanji M Arbustini E Stuppia L Ceriello L Girolamo E Is occult genetic substrate the missing link between arrhythmic mitral annular disjunction syndrome and sudden cardiac death? *Can J Cardiol.* (2021) 37:1651–3. 10.1016/j.cjca.2021.04.014 33933609

[62] Giudicessi J Maleszewski J Tester D Ackerman M. Prevalence and potential genetic determinants of young sudden unexplained death victims with suspected arrhythmogenic mitral valve prolapse syndrome. *Heart Rhythm O2.* (2021) 2:431–8. 10.1016/j.hroo.2021.07.006 34667957 PMC8505213

[63] Giudicessi J Rohatgi R Bos J Ackerman M. Prevalence and clinical phenotype of concomitant long QT syndrome and arrhythmogenic bileaflet mitral valve prolapse. *Int J Cardiol.* (2019) 274:175–8. 10.1016/j.ijcard.2018.09.046 30219255

[64] Oyama M Elliott C Loughran K Kossar A Castillero E Levy R Comparative pathology of human and canine myxomatous mitral valve degeneration: 5HT and TGF-β mechanisms. *Cardiovasc Pathol.* (2020) 46:107196. 10.1016/j.carpath.2019.107196 32006823 PMC7078050

[65] O’Brien M Beijerink N Wade C. Genetics of canine myxomatous mitral valve disease. *Anim Genet.* (2021) 52:409–21. 10.1111/age.13082 34028063

[66] Meurs K Friedenberg S Williams B Keene B Atkins C Adin D Evaluation of genes associated with human myxomatous mitral valve disease in dogs with familial myxomatous mitral valve degeneration. *Vet J.* (2018) 232:16–9. 10.1016/j.tvjl.2017.12.002 29428085

[67] Oyama M Chittur S. Genomic expression patterns of mitral valve tissues from dogs with degenerative mitral valve disease. *Am J Vet Res.* (2006) 67:1307–18. 10.2460/ajvr.67.8.1307 16881841

[68] Cremer S Singletary G Olsen L Wallace K Häggström J Ljungvall I Serotonin concentrations in platelets, plasma, mitral valve leaflet, and left ventricular myocardial tissue in dogs with myxomatous mitral valve disease. *J Vet Intern Med.* (2014) 28:1534–40. 10.1111/jvim.12420 25146933 PMC4895588

[69] Madsen M Olsen L Häggström J Höglund K Ljungvall I Falk T Identification of 2 loci associated with development of myxomatous mitral valve disease in Cavalier King Charles Spaniels. *J Hered.* (2011) 102(Suppl. 1):S62–7. 10.1093/jhered/esr041 21846748

[70] Motenko H Neuhauser S O’Keefe M Richardson J. MouseMine: a new data warehouse for MGI. *Mamm Genome.* (2015) 26:325–30. 10.1007/s00335-015-9573-z 26092688 PMC4534495

[71] Feng Y Chen M Moskowitz I Mendonza A Vidali L Nakamura F Filamin A (FLNA) is required for cell-cell contact in vascular development and cardiac morphogenesis. *Proc Natl Acad Sci U.S.A.* (2006) 103:19836–41. 10.1073/pnas.0609628104 17172441 PMC1702530

[72] Sauls K Toomer K Williams K Johnson A Markwald R Hajdu Z Increased infiltration of extra-cardiac cells in myxomatous valve disease. *J Cardiovasc Dev Dis.* (2015) 2:200–13. 10.3390/jcdd2030200 26473162 PMC4603574

[73] Remy S Chenouard V Tesson L Usal C Ménoret S Brusselle L Generation of gene-edited rats by delivery of CRISPR/Cas9 protein and donor DNA into intact zygotes using electroporation. *Sci Rep.* (2017) 7:16554. 10.1038/s41598-017-16328-y 29185448 PMC5707420

[74] Chow R Vermot J. The rise of photoresponsive protein technologies applications in vivo: a spotlight on zebrafish developmental and cell biology. *F1000Research.* (2017) 6:F1000 Faculty Rev-459. 10.12688/f1000research.10617.1 28413613 PMC5389412

[75] Moskowitz I Wang J Peterson M Pu W Mackinnon A Oxburgh L Transcription factor genes Smad4 and Gata4 cooperatively regulate cardiac valve development. [corrected]. *Proc Natl Acad Sci U.S.A.* (2011) 108:4006–11. 10.1073/pnas.1019025108 21330551 PMC3053967

[76] Connolly H Crary J McGoon M Hensrud D Edwards B Edwards W Valvular heart disease associated with fenfluramine-phentermine. *N Engl J Med.* (1997) 337:581–8. 10.1056/NEJM199708283370901 9271479

[77] Djenoune L Berg K Brueckner M Yuan S. A change of heart: new roles for cilia in cardiac development and disease. *Nat Rev Cardiol.* (2022) 19:211–27. 10.1038/s41569-021-00635-z 34862511 PMC10161238

[78] Morningstar J Gensemer C Moore R Fulmer D Beck T Wang C Mitral valve prolapse induces regionalized myocardial fibrosis. *J Am Heart Assoc.* (2021) 10:e022332. 10.1161/JAHA.121.022332 34873924 PMC9075228

[79] Lin C Lin C Chen C Zhou B Chang C. Partitioning the heart: mechanisms of cardiac septation and valve development. *Development.* (2012) 139:3277–99. 10.1242/dev.063495 22912411 PMC3424040

[80] Christoffels V Hoogaars W Tessari A Clout D Moorman A Campione M. T-box transcription factor Tbx2 represses differentiation and formation of the cardiac chambers. *Dev Dyn.* (2004) 229:763–70. 10.1002/dvdy.10487 15042700

[81] Moorman A Webb S Brown N Lamers W Anderson R. Development of the heart: formation of the cardiac chambers and arterial trunks. *Heart.* (2003) 89:806–14. 10.1136/heart.89.7.806 12807866 PMC1767747

[82] Person A Klewer S Runyan R. Cell biology of cardiac cushion development. *Int Rev Cytol.* (2005) 243:287–335. 10.1016/S0074-769643005-3 15797462

[83] Dal-Bianco J Aikawa E Bischoff J Guerrero J Handschumacher M Sullivan S Active adaptation of the tethered mitral valve: insights into a compensatory mechanism for functional mitral regurgitation. *Circulation.* (2009) 120:334–42. 10.1161/CIRCULATIONAHA.108.846782 19597052 PMC2752046

[84] Shapero K Wylie-Sears J Levine R Mayer J Bischoff J. Reciprocal interactions between mitral valve endothelial and interstitial cells reduce endothelial-to-mesenchymal transition and myofibroblastic activation. *J Mol Cell Cardiol.* (2015) 80:175–85. 10.1016/j.yjmcc.2015.01.006 25633835 PMC4346432

[85] Wylie-Sears J Levine R Bischoff J. Losartan inhibits endothelial-to-mesenchymal transformation in mitral valve endothelial cells by blocking transforming growth factor-β-induced phosphorylation of ERK. *Biochem Biophys Res Commun.* (2014) 446:870–5. 10.1016/j.bbrc.2014.03.014 24632204 PMC4007266

[86] Lu C Liu M Clinton M Culshaw G Argyle D Corcoran B. Developmental pathways and endothelial to mesenchymal transition in canine myxomatous mitral valve disease. *Vet J.* (2015) 206:377–84. 10.1016/j.tvjl.2015.08.011 26586213

[87] Kim A Alfieri C Yutzey K. Endothelial cell lineage analysis does not provide evidence for EMT in adult valve homeostasis and disease. *Anat Rec.* (2019) 302:125–35. 10.1002/ar.23916 30306735 PMC6312497

[88] Tang Q McNair A Phadwal K Macrae V Corcoran B. The role of transforming growth factor-β signaling in myxomatous mitral valve degeneration. *Front Cardiovasc Med.* (2022) 9:872288. 10.3389/fcvm.2022.872288 35656405 PMC9152029

[89] Bartko P Dal-Bianco J Guerrero J Beaudoin J Szymanski C Kim D Effect of losartan on mitral valve changes after myocardial infarction. *J Am Coll Cardiol.* (2017) 70:1232–44. 10.1016/j.jacc.2017.07.734 28859786 PMC5679094

[90] Dal-Bianco J Aikawa E Bischoff J Guerrero J Hjortnaes J Beaudoin J Myocardial infarction alters adaptation of the tethered mitral valve. *J Am Coll Cardiol.* (2016) 67:275–87. 10.1016/j.jacc.2015.10.092 26796392 PMC5099077

[91] Hulin A Moore V James J Yutzey K. Loss of Axin2 results in impaired heart valve maturation and subsequent myxomatous valve disease. *Cardiovasc Res.* (2017) 113:40–51. 10.1093/cvr/cvw229 28069701 PMC5220675

[92] Butcher J Penrod A García A Nerem R. Unique morphology and focal adhesion development of valvular endothelial cells in static and fluid flow environments. *Arterioscler Thromb Vasc Biol.* (2004) 24:1429–34. 10.1161/01.ATV.0000130462.50769.5a 15117733

[93] Merryman W Youn I Lukoff H Krueger P Guilak F Hopkins R Correlation between heart valve interstitial cell stiffness and transvalvular pressure: implications for collagen biosynthesis. *Am J Physiol Heart Circ Physiol.* (2006) 290:H224–31. 10.1152/ajpheart.00521.2005 16126816

[94] Duval D Labbé P Bureau L Le Tourneau T Norris R Markwald R MVP-associated Filamin A mutations affect FlnA-PTPN12 (PTP-PEST) interactions. *J Cardiovasc Dev Dis.* (2015) 2:233–47. 10.3390/jcdd2030233 26594644 PMC4654411

[95] Blomme B Deroanne C Hulin A Lambert C Defraigne J Nusgens B Mechanical strain induces a pro-fibrotic phenotype in human mitral valvular interstitial cells through RhoC/ROCK/MRTF-A and Erk1/2 signaling pathways. *J Mol Cell Cardiol.* (2019) 135:149–59. 10.1016/j.yjmcc.2019.08.008 31442470

[96] Wilcken D Hickey A. Lifetime risk for patients with mitral valve prolapse of developing severe valve regurgitation requiring surgery. *Circulation.* (1988) 78:10–4. 10.1161/01.cir.78.1.10 3383395

